# Exploring Psychotherapy Approaches for Dyspnea: A Systematic Review Protocol

**DOI:** 10.1089/pmr.2025.0002

**Published:** 2025-02-21

**Authors:** Jun Kako, Kohei Kajiwara, Masamitsu Kobayashi, Yoshiyasu Ito, Kanako Ichikura, Yoshinobu Matsuda, Takashi Yamaguchi

**Affiliations:** ^1^ Graduate School of Medicine, Mie University, Tsu, Japan.; ^2^ Faculty of Nursing, Japanese Red Cross Kyushu International College of Nursing, Munakata, Japan.; ^3^ Graduate of Nursing Science, St. Luke’s International University, Chuo-ku, Japan.; ^4^ Faculty of Nursing Science, Tsuruga Nursing University, Tsuruga, Japan.; ^5^ School of Allied Health Sciences, Kitasato University, Sagamihara, Japan.; ^6^ Department of Psychosomatic Internal Medicine, NHO Kinki Chuo Chest Medical Center, Sakai, Japan.; ^7^ Department of Palliative Medicine, Kobe University Graduate School of Medicine, Kobe, Japan.

**Keywords:** cancer, dyspnea, noncancer, psychotherapy, systematic review

## Abstract

**Introduction:**

Dyspnea, a common and debilitating symptom, affects over half of the patients with cancer, with increasing frequency and severity as the end of life approaches. It substantially affects the daily lives of patients by contributing to anxiety, depression, fatigue, and reduced physical activity, ultimately diminishing their quality of life. Although pharmacological treatments remain standard, nonpharmacological interventions, including psychotherapy, are increasingly recommended owing to their safety and accessibility. Psychotherapy is particularly relevant for alleviating psychological distress associated with dyspnea; however, research on its efficacy in managing dyspnea among patients with cancer is limited. This systematic review aims to synthesize evidence on the following four key psychotherapy techniques for managing dyspnea: mindful breathing, guided imagery, progressive muscle relaxation, and meditation. The review will assess the optimal delivery methods, evaluate the quality of existing studies, and provide clinical recommendations for implementing these interventions in cancer care.

**Method and Analysis:**

This review adheres to the Preferred Reporting Items for Systematic Reviews and Meta-Analyses protocols. A systematic search will be conducted on some databases such as PubMed. Relevant studies will also be identified through reference lists and manual searches of key journals. The search terms will include keywords related to dyspnea and psychotherapeutic techniques. Four independent reviewers will screen and assess the articles, extracting data using a standardized charting form. The quality of the studies will be assessed using the Mixed Methods Assessment Tool. The results will be summarized via a psychotherapeutic technique, providing a comprehensive overview of the interventions and their applicability.

## Introduction

Dyspnea affects over half of the patients with cancer, with increasing frequency and severity as the time of death approaches.^1–4^ -Moreover, its impact on daily life is profound, encompassing increased anxiety, depression, loss of appetite, fatigue, and decreased quality of life, as well as reduced motivation to live and limited physical activity.^5–10^ Therefore, the effective management of dyspnea is critical.

The managing strategies of dyspnea include both pharmacological and nonpharmacological interventions. Recent clinical guidelines prioritize nonpharmacological interventions owing to their high safety profile and convenience.^11,12^ Evidence supporting nonpharmacological interventions for dyspnea, particularly fan therapy,^13–19^ is increasing, with this approach strongly recommended in clinical guidelines.^11,12^ However, research on other nonpharmacological interventions is limited.

Dyspnea is associated with psychological symptoms, such as anxiety and depression.^20^ This association can be explained using the Breathing, Thinking, and Functioning model, which provides a framework for understanding dyspnea.^21,22^ It conceptualizes emotional and behavioral responses to dyspnea, as well as the vicious cycle that can occur. The model comprises three domains as follows: breathing, thinking, and functioning. Nonpharmacological interventions in the breathing domain include fan therapy, breathing techniques, and inspiratory muscle training. In the thinking domain, mindfulness, relaxation, and meditation are applicable. In the functioning domain, interventions such as breathing rehabilitation, activity promotion, use of walking aids, and pacing are recommended.^21,22^ Dyspnea is associated with feelings of anxiety, fear, and tension; thus, interventions, such as psychotherapy, may be applied in the thinking domain.

Although a few studies specifically reported the effectiveness of psychotherapy for dyspnea in patients with cancer, it is often included as part of a complex intervention.^23^ Psychotherapy is often used as a form of support in clinical practice, and its^24^ effectiveness in addressing mental symptoms, such as anxiety and depression, has been well documented. Moreover, it may be effective for managing dyspnea.^25–28^ Therefore, our research group is exploring the development of an application (app), highly accessible and easy to implement, to deliver psychotherapy for dyspnea, which will focus on four psychotherapeutic techniques—mindful breathing,^29,30^ guided imagery,^31–35^ progressive muscle relaxation,^31,32,36,37^ and meditation^38^—particularly suited for this purpose. A major challenge with psychotherapy is its high heterogeneity, arising from variations in techniques, delivery methods, and content.^39^ Therefore, ensuring a unified approach for delivering each type of psychotherapy via an app is important. However, research on these psychotherapies for breathing difficulties experienced by patients with cancer is limited.^23^

Therefore, in this review, to explore the optimal methods of providing each psychotherapy offered by the app, we will first conduct a comprehensive literature review that is not limited to cancer but includes a wide range of noncancer conditions. Second, by evaluating the quality of the literature extracted for each psychotherapy, we will identify recommendations for higher-quality provision methods. Third, we will organize the current situation and issues of each psychotherapy for dyspnea in patients with cancer from studies limited to patients with cancer and obtain suggestions for clinical practice.

## Objectives

The proposed review will comprehensively investigate mindfulness breathing, guided imagery, progressive muscle relaxation, and meditation for managing dyspnea. Moreover, it seeks to identify the methodology of each technique and evaluate the quality of evidence for these interventions. Where feasible, a subgroup analysis will be conducted focusing on patients with cancer.

### Study design

This study is a systematic review.

## Methodology

### Study design and registration

This systematic review adheres to the Preferred Reporting Items for Systematic Reviews and Meta-Analyses Protocols (PRISMA-P) guidelines^40^ to ensure comprehensive and transparent reporting. The checklist is included as Supplementary Data S1. The final review will be reported in accordance with the PRISMA guidelines.^41,42^ This study was registered before data extraction with the University Hospital Medical Information Network, Japan (UMIN 000056589).

### Study eligibility criteria

The initial aim of this study is to develop accessible apps for mindful breathing, guided imagery, progressive muscle relaxation, and meditation for dyspnea in patients with cancer. The aim also includes identifying methodologies and evaluating the effectiveness of each technique. However, a preliminary PubMed search before this study on December 31, 2024, revealed that the number of studies on psychological therapy was extremely limited, with <120 articles. Following discussions with the research team, the scope of the review was expanded to include noncancer conditions rather than restricting it to cancer.

The following eligibility criteria were established by a multidisciplinary team of researchers, including doctors, nurses, and psychologists with experience in dyspnea, psychotherapy, and systematic reviews. Throughout the screening and data extraction processes, these criteria will be discussed within the research team and updated as needed to ensure the inclusion of all relevant literature.

*Inclusion criteria*

The following inclusion criteria will be applied:

- Patients/Participants
  – Patients with dyspnea
  – Patients aged 18 years or older
- Intervention
  – Mindful breathing, guided imagery, progressive muscle relaxation, and meditation in all patients
- Comparison
  – None in particular
- Outcomes
  – Only quantitative data will be considered acceptable
- Study design
  – Randomized controlled trial (RCT), non-RCT, crossover trial, single-arm pre-/post-comparative study, cohort study (prospective or retrospective), case series, and case reports
- Articles written in English

*Exclusion criteria*

The following exclusion criteria will be applied:

- Patients/Participants
  – None in particular
- Intervention
  – None in particular
- Comparison
  – None in particular
- Outcomes
  – None in particular
- Study design
  – Review article (systematic review/meta-analysis), response to letter, opinion (expert opinion), study protocol, conference proceedings or abstract, cross-sectional study, books or book review, and qualitative research (such as observations, interviews, and focus groups).

The study designs listed above were excluded because they do not provide original or primary research data directly related to the research objectives. These designs, such as review articles, expert opinions, and study protocols, typically summarize or interpret existing data rather than generating new empirical evidence. In addition, qualitative studies such as interviews and focus groups, while valuable, may not align with the methodological requirements of this review or analysis, which prioritizes studies with quantifiable or replicable outcomes.

### Search strategy

We will systematically search all published articles using the following electronic databases: PubMed, CINAHL, the Cochrane Central Register of Controlled Trials in the Cochrane Library, and Scopus. In addition, we will examine relevant studies from the reference lists and manually search major journals.

In developing the search strategy, researchers with expertise in dyspnea, psychotherapy, and systematic reviews will identify relevant keywords. The final version of the search strategy will first be applied to the PubMed database (Supplementary Data S2) and subsequently adapted for use in other databases.

### Study selection

In this study, we will examine mindful breathing, guided imagery, progressive muscle relaxation, and meditation. A meeting will be held to discuss the preliminary inclusion and exclusion criteria during the protocol development phase. At least four reviewers will refine the search strategy based on retrieved articles. The reviewers will meet at the beginning, midpoint, and conclusion of the article review process to address any issues or uncertainties related to the selection of studies and modify the search strategy as necessary.

For the first screening, the titles and abstracts of all references obtained through database searches will be independently reviewed by four reviewers using the Rayyan data management software. During the second screening, the full texts of the references selected during the first screening will be independently reviewed by four reviewers. An article will be included if it meets the eligibility criteria and does not fall under any of the exclusion criteria. If the relevance of a study is unclear during the screening process or if disagreements arise between the four reviewers, a meeting will be held to resolve the discrepancies. If a consensus cannot be reached, a fifth reviewer will be consulted to make the final decision.

### Data extraction

We will develop a data chart for extracting and recording variables relevant to the research question. The data extraction process will then be piloted using 5–10 selected articles to ensure comprehensive and accurate data collection. The procedures will be discussed, and two reviewers will perform the full data extraction. Another researcher will review all the data extracted from all articles to identify discrepancies and ensure the reliability of the process.

A preliminary data-extraction framework has been developed to address the predefined research questions. In addition to basic bibliographic information (such as the first author name, year of publication, location, or country of study), the following information will be recorded: (1) study design, setting, and sample size; (2) age, sex, disease, intensity of dyspnea; (3) specific methods used to deliver each psychological therapy (such as provider, duration, and frequency of implementation); (4) outcome assessment tools, assessment time points, and frequency; and (5) details of the observed effects.

### Risk of bias

We will use the Mixed Methods Assessment Tool (MMAT) to qualitatively assess the studies included in this review.^43^ The MMAT is designed for common research methods and designs and is widely used across various fields, including health sciences, education, information science, and psychology. Each study will be rated based on the number of criteria met as follows: 0% (very poor quality), no criteria met; 25% (low quality), one criterion met; 50% (moderate quality), two criteria met; 75% (considerable quality), three criteria met; and 100% (high quality), all criteria met. Four reviewers will independently assess the quality of selected studies. In cases of disagreements, all reviewers will discuss the issue until a consensus is reached. A fifth reviewer will make the final decision if a consensus cannot be achieved.

### Data analysis and synthesis

For each included study, we will summarize the basic bibliographic information, research design, sample size, demographic data, and type of psychological therapy used. The methods used to deliver each type of psychological therapy, including provider, duration, and frequency, will be presented in a table.

In addition, we will examine the psychological therapy approaches used specifically for patients with cancer. For each type of psychological therapy (e.g., mindful breathing, guided imagery, progressive muscle relaxation, meditation), we will describe the delivery methods, including the provider, duration, frequency, and any other relevant characteristics. This will help to provide a comprehensive understanding of how each psychological therapy intervention is applied within this population.

### Safety consideration

This section does not apply, as this is a systematic review.

### Follow-up process

This section does not apply, as this is a systematic review.

## Data Availability

Data sharing is not applicable as no datasets were generated and/or analyzed for this study.
