# Supplementary material for: Exploring Psychotherapy Approaches for Dyspnea: A Systematic Review Protocol
Source: Palliat Med Rep. 2025 Feb 21;6(1):84–9. doi: 10.1089/pmr.2025.0002 (PMC11947646; doi:10.1089/pmr.2025.0002)
Supplement: Supplementary Data S2 [file pmr.2025.0002_supplementary_datas2.docx]

Supplementary file 2. Search stretegy used in PubMed database

| #1 Dyspnea | "dyspnea"[Title/Abstract] OR "dyspnoea"[Title/Abstract] OR "dyspneic"[Title/Abstract] OR "breathless"[Title/Abstract] OR "breathlessness"[Title/Abstract] OR "short of breath"[Title/Abstract] OR "shortness of breath"[Title/Abstract] OR "breathing difficulty"[Title/Abstract] OR "labored breathing"[Title/Abstract] |
| --- | --- |
| #2 Mindfulness breathing | "Breathing Exercises"[MeSH Terms] OR "Breathing Exerci*"[Title/Abstract] OR "mindful breath*"[Title/Abstract] OR "breathing techniq*"[Title/Abstract] OR "mindfulness"[MeSH Terms] OR "mindful*"[Title/Abstract] OR "mind train*"[Title/Abstract] OR "MBCT"[Title/Abstract] |
| #3 Guided imagery | "imagery, psychotherapy"[MeSH Terms] OR ("imagery"[Title/Abstract] AND "psychotherapy"[Title/Abstract]) OR "imagery"[Title/Abstract] OR "Mindful*"[MeSH Terms] OR "guided imager*"[Title/Abstract] OR "imagery therap*"[Title/Abstract] OR "guided imagery therap*"[Title/Abstract] OR "mental imager*"[Title/Abstract] |
| #4 Progressive muscle relaxation | "progressive muscle relaxation"[Title/Abstract] OR "PMR"[Title/Abstract] OR "muscle relaxation"[Title/Abstract] OR "relaxation techniq*"[Title/Abstract] OR "relaxation therap*"[Title/Abstract] OR "Progressive Relaxation"[Title/Abstract] |
| #5 Meditation | "meditation"[MeSH Terms] OR "meditation*"[Title/Abstract] OR "mindful*"[Title/Abstract] OR "mindfulness meditation"[Title/Abstract] OR "mindfulness-based stress reduction"[Title/Abstract] OR "mindfulness-based breathing therap*"[Title/Abstract] OR "meditative practic*"[Title/Abstract] OR "mediation techniq*"[Title/Abstract] OR "mediation practic*"[Title/Abstract] |
| #6 | #1 AND (#2 OR #3 OR #4 OR #5) |
